# Recent advances in polysaccharides from *Tremella fuciformis*: isolation, structures, bioactivities and application

**DOI:** 10.3389/fnut.2025.1663327

**Published:** 2025-11-19

**Authors:** Siyu Li, Ke Zhao, Jinjun Li, Xiaoqiong Li, Hui Zhao, Ruyan Cui, Jianmei Li, Jinbin Guo, Xiangyu Bian

**Affiliations:** 1 Zhejiang Key Laboratory of Intelligent Food Logistics and Processing, Institute of Food Science, Zhejiang Academy of Agricultural Sciences, Hangzhou, China; 2 State Key Laboratory for Molecular and Developmental Biology, Institute of Genetics and Developmental Biology, Chinese Academy of Sciences, Beijing, China; 3 Department of Otolaryngology Head and Neck Surgery, Tangdu Hospital, Air Force Medical University, Xi'an, China; 4 Affiliated Hospital of Changzhi Institute of Traditional Chinese Medicine, Changzhi, China

**Keywords:** *Tremella fuciformis* polysaccharides, structure–activity relationship, pharmacological action, functional foods, applications

## Abstract

*Tremella fuciformis*, commonly known as snow fungus, is a traditional edible and medicinal fungus widely used in Asian countries for centuries. Its main bioactive constituents, *Tremella fuciformis* polysaccharides (TFPs), have attracted considerable attention in both food and pharmaceutical industries due to their diverse biological activities. TFPs exhibit a broad spectrum of health-promoting effects, including microbial homeostasis, immuno-modulatory, antioxidant, anti-hyperglycemic, anti-hyperlipidemia, and others. This review summarizes recent advances in the extraction, purification, structural characterization, and pharmacological action of TFPs. The findings suggest that TFPs are promising natural bioactive compounds with potential applications in disease prevention and treatment, and they represent valuable candidates for the development of functional foods and therapeutic agents.

## 1 Introduction

*Tremella fuciformis*, an edible and medicinal fungus, possess notable medicinal properties and bioactivities in its fruiting bodies. It belongs to the order *Tremellaces* and the family *Tremellaceae*, exhibiting numerous clusters of flat, flaky or wavy leaflets (1). *Tremella fuciformis* is known as “silver fungus” or “white fungus” in China. The *Tremella fuciformis* was first mentioned in Shennong Bencao Jing (Shennong’s Classic of Materia Medica; 200–300AD). For thousands of years, *Tremella fuciformis* has been regarded as a precious traditional medicine for nourishing the stomach and lungs, and improving weakness.

*Tremella fuciformis* contains a variety of bioactive ingredients, including fatty acids, proteins, enzymes, polysaccharides, phenols, flavonoids, dietary fibers, and trace elements (2–4). Among which the most important polysaccharides extracted from *Tremella fuciformis* were considered as the main active components. Based on their origin and structural features, *Tremella fuciformis* polysaccharides (TFPs) are generally classified into five major categories: acidic heteropolysaccharides, neutral heteropolysaccharides, acidic oligosaccharides, cell-wall polysaccharides, and exopolysaccharides. Reported molecular weights for different TFPs span a broad range, from 1.08 × 10^3^ Da to 3.74 × 10^6^ Da. Notably, numerous *in vitro* studies have suggested that low-molecular-weight TFPs exhibit higher antioxidant activity compared to their high-molecular-weight counterparts. However, *in vitro* models cannot adequately replicate the complex physiological conditions within a living organism. Given the stark differences between data from in vitro assays and the biochemical environment *in vivo*, investigation using mammalian models is imperative. Such studies are essential to definitively elucidate the structure–activity relationships of TFPs and thereby accelerate their development as functional foods and therapeutic agents.

Significant progress has been made in understanding the chemical and biological activities of TFPs in recent decades. Numerous studies have shown that TFP_S_ exhibits diverse physiological and health-promoting effects, including antioxidant (5–10), antitumor (11–15), immunomodulation (16–21), anti-inflammation (22–25), gastroprotective (26–28), hepatoprotective (29), neuroprotective (30–35), antidiabetic (36–39), anti-obesity (40, 41), anti-radiation (42, 43), and drug delivery (44, 45) activities. In 2002, after been approval by Chinese Food and Drug Administration (CFDA), “*Tremella Fuciformis* polysaccharide enteric capsules” derived from TFPs were introduced to alleviate leukopenia symptoms in cancer patients undergoing chemotherapy and radiotherapy (46–50). Additionally, these capsules are widely used as adjuvant therapies for chronic persistent hepatitis, chronic active hepatitis, mycoplasma pneumonia, and subjective cognitive disorders and other conditions (30, 51–53). Medicines derived from TFPs are primarily based on the natural active component glycosyl, offering broad applications, low toxicity, and minimal drug resistance. These characteristics provide a significant advantage over synthetic medicines. Furthermore, TFPs exhibit minimal side effects and are cost-effective, offering significant potential for future applications (54). Therefore, a comprehensive investigation into the physicochemical properties and biological activities of TFPs is crucial.

Although numerous research articles address edible fungi, literature reviews specifically focused on TFPs remain scarce. This study reviews recent advancements in the extraction and purification methods, structural characterization, and biological activities of TFPs, aiming to provide a comprehensive reference for its applications in functional ingredient, flavoring, food, and pharmaceutical industry.

## 2 Extraction and purification methods

*Tremella fuciformis* polysaccharides, a class of microbial glycans, can be extracted from the spores, mycelium, or fermentation broth of *Tremella fuciformis*. They are primarily composed of acidic heteropolysaccharides, neutral heteropolysaccharides, acidic oligosaccharides, cell-wall polysaccharides, and exopolysaccharides. The backbone consists mainly of mannan, with side chains linked at the 2, 4, and 6 positions by glucose, xylose, fucose, and galactose residues. TFPs typically have a molecular weight ranging from 1.3 to 1.8 million Da. Their yield, structural integrity, and biological activity are highly dependent on the extraction process (55). The extraction yield of a TFPs depends on several factors. Structural complexity-TFPs have higher molecular weights, intricate tertiary and quaternary structures, and lower solubility compared to other fungal polysaccharides. Physical properties-in aqueous solution, TFPs adopt an extended conformation and exhibit high viscosity, complicating extraction and purification. Therefore, beyond maximizing yield, preserving structural integrity is essential, making the selection of appropriate extraction methods critical for ensuring bioactivity in subsequent applications.

Common extraction methods for TFPs include hot-water extraction, alkali or acid extraction, ultrasound-assisted extraction (often with polyethylene glycol), and enzymatic hydrolysis. Combining two or more methods can increase TFPs yield by 3–4 fold (56). Among these, hot-water extraction is the most widely used approach (6, 9, 16, 31, 34, 43, 57–59), often combined with auxiliary techniques such as freeze–thaw cycles (6, 34, 35), microwave (60), or ultrasound assistance (1, 61). This method avoids the use of acid, alkali, or organic solvents, making it environmentally friendly and simplifying downstream processing. Consequently, it has become a classical and industrially preferred method for TFPs preparation. However, hot-water extraction typically requires high temperatures and prolonged durations, which can lead to polysaccharide degradation and alter their structural and functional properties. To minimize these effects, extraction is recommended at 90 °C for no more than 5 h (6, 27, 62). Vacuum concentration further helps preserve bioactivity during processing.

Crude aqueous extracts can be purified by ethanol precipitation, gradient fractionation, ion exchange chromatography, gel filtration, and affinity chromatography (63). Gel filtration separates polysaccharides based on molecular weight, with higher molecular weight fractions eluting earlier. Jin et al. used DEAE-52 anion-exchange chromatography followed by Sepharose G-100 gel filtration to isolate a fraction (TL04) with a molecular weight of 2.0 × 10^6^ Da (31). Similarly, Ge et al. employed DEAE-Sepharose ion-exchange chromatography to separate neutral and acidic polysaccharides from fermentation broth, yielding two distinct fractions: TFPA (2.3 × 10^5^ Da) and TFPB (1.1 × 10^5^ Da) (7). TFPB was also isolated using a dialysis membrane with a 3.5 × 10^4^ Da cutoff.

**Figure 1:**
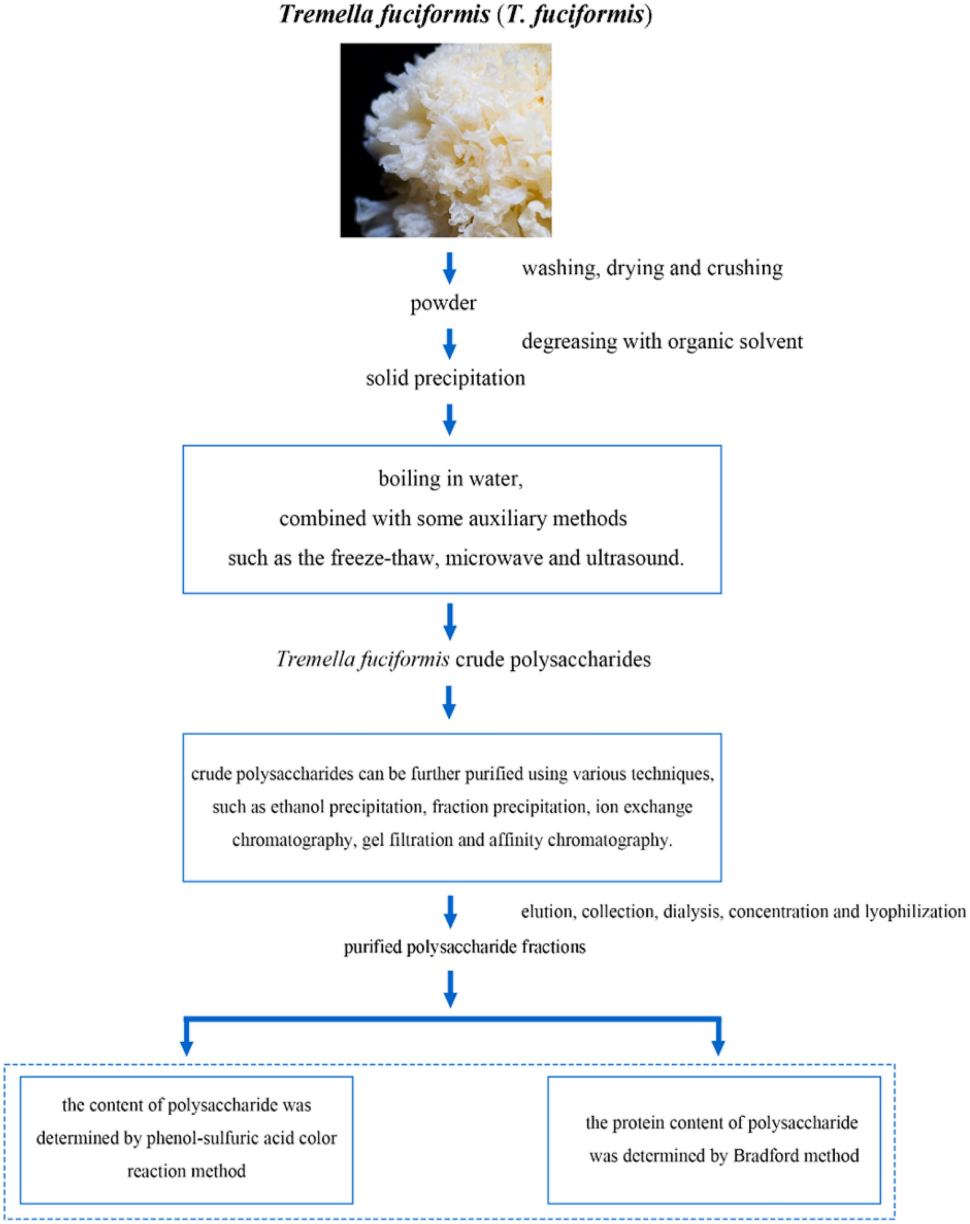
The flow chart of extraction and purification of *Tremella fuciformis* polysaccharides.

In summary, the extraction of TFPs typically begins with washing the fruiting bodies with distilled water to remove impurities, followed by drying and grinding into powder. The powdered sample is then suspended in water and heated in a water bath. After filtration and centrifugation, the supernatant is collected. To precipitate the polysaccharides, ethanol is added, and the mixture is stirred and refrigerated at 4 °C overnight. The resulting precipitate is centrifuged, dried, and ground to yield crude TFP powder. The crude extract is subsequently redissolved and purified through various chromatographic techniques, including ion exchange and gel filtration chromatography. The eluted fractions are collected, dialyzed, concentrated, and lyophilized to obtain purified polysaccharide fractions. The polysaccharide content is quantified using the phenol-sulfuric acid colorimetric method, while residual protein is determined by the Bradford assay. A schematic overview of the extraction and purification workflow is provided in Figure 1.

## 3 Physicochemical and structural features

The particle size, glycosidic bonds, chain conformation, degree of branching, molecular weight and chemical composition of polysaccharides significantly affect their natural biological activity. To date, a total of 38 polysaccharides has been identified from *Tremella fuciformis*. Their main structural features, such as monosaccharide composition and molecular weight, are shown in Table 1, along with their names and corresponding references are included.

**Table 1:** The polysaccharides isolated from *Tremella fuciformis.*

No.	Compound name	Nature of the polysaccharide	*Monosaccharide composition*	Structures	Molecular weight (kDa)	Extraction method	Pharmacological properties	References
1	HWS-A	Acidic heteropolysaccharide	D-Xyl, D-Man, D-GlcA in the ratio of 1.0:2.9:1.0	α-(1 → 3)-linked D-Man backbone chain to which *β*-(1 → 2)-linked D-GlcA and single or short chains of β-(1 → 2)-linked D-Xyl residues are attached at the C-2 position		Alkali extraction	Immunomodulation	(64)
2	HAS-A	Acidic heteropolysaccharide	D-Xyl, D-Man, D-GlcA in the ratio of 1.0:4.9:1.3	α-(1 → 3)-linked D-Man backbone chain to which β-(1 → 2)-linked D-GlcA and single or short chains of β-(1 → 2)-linked D-Xyl residues are attached at the C-2 position		Alkali extraction	Immunomodulation	(64)
3	TFBP-A	Acidic heteropolysaccharide	Man, Gal, Glc in the ratio of 90:5:5	Not reported	59	Alkali extraction	Antioxidant	(65)
4	T1a	Heteropolysaccharide	Man, Xyl, Glc, Fuc and GlcA	Consisted of (1 → 3)-linked Man, which was branched at the 2, 4, or 6 positions	53	Hot-water extraction	Immunomodulation	(21)
5	T1b	Heteropolysaccharide	Man, Xyl, Glc, Fuc and GlcA	Consisted of (1 → 3)-linked Man, which was branched at the 2, 4, or 6 positions	18	Hot-water extraction	Immunomodulation	(21)
6	T1c	Heteropolysaccharide	Man, Xyl, Glc, Fuc and GlcA	Consisted of (1 → 3)-linked Man, which was branched at the 2, 4, or 6 positions	12	Hot-water extraction	Immunomodulation	(21)
7	T2a	Acidic heteropolysaccharide	Man, GlcA, Xyl, Glc and Fuc	A Man back bone consisting of 3-linked Man, and side chains containing glucosyl, Man, Fuc, Xyl, and GlcA residues	410		Immunomodulation	(19)
8	T2b	Acidic heteropolysaccharide	Man, GlcA, Xyl, Glc and Fuc	A Man back bone consisting of 3-linked Man, and side chains containing glucosyl, Man, Fuc, Xyl, and GlcA residues	250		Immunomodulation	(19)
9	T2c	Acidic heteropolysaccharide	Man, GlcA, Xyl, Glc and Fuc	A Man back bone consisting of 3-linked Man, and side chains containing glucosyl, Man, Fuc, Xyl, and GlcA residues	34		Immunomodulation	(19)
10	T2d	Acidic heteropolysaccharide	Man, GlcA, Xyl, Glc and Fuc	A Man back bone consisting of 3-linked Man, and side chains containing glucosyl, Man, Fuc, Xyl, and GlcA residues	20		Immunomodulation	(19)
11	T3a	Acidic heteropolysaccharide	Man, Glc, Xyl, GlcA and Fuc	a Man backbone consisting of 3-linked Manp, and side chains containing Glc, Man, Fuc, Xyl, and GlcA residues attached through 0–2, 0–4, or 0–6 of about half of the backbone Man residues	550	Hot-water extraction	Immunomodulation	(20)
12	T3b	Acidic heteropolysaccharide	Man, Glc, Xyl, GlcA and Fuc	a Man backbone consisting of 3-linked Manp, and side chains containing Glc, Man, Fuc, Xyl, and GlcA residues attached through 0–2, 0–4, or 0–6 of about half of the backbone Man residues	420	Hot-water extraction	Immunomodulation	(20)
13	T3c	Acidic heteropolysaccharide	Man, Glc, Xyl, GlcA and Fuc	a Man backbone consisting of 3-linked Manp, and side chains containing Glc, Man, Fuc, Xyl, and GlcA residues attached through 0–2, 0–4, or 0–6 of about half of the backbone Man residues	55	Hot-water extraction	Immunomodulation	(20)
14	T3d	Acidic heteropolysaccharide	Man, Glc, Xyl, GlcA and Fuc	a mannan backbone consisting of 3-1inked Manp, and side chains containing glucosyl, mannosyl, fucosyl, xylosyl, and glucuronic acid residues attached through 0–2, 0–4, or 0–6 of about half of the backbone mannosyl residues	48	Hot-water extraction	Immunomodulation	(20)
15	WTF-B		Glc, Man in the ratio of 8:2	the backbone was composed of (1 → 3)-linked β-D-mannopyranoside, and the side chain composed of (1 → 6)-linked β-D-xylopyranoside was attached to the C2 of the backbone mannopyranoside	68	Hot-water extraction	radiation protection	(43)
16	TP-1		Man, Xyl, GlcA in the ratio of 60. 5:18.9:20.6	the backbone was composed of (1 → 3)-linked β-D-mannopyranoside, and the side chain composed of (1 → 6)-linked β-D-xylopyranoside was attached to the C2 of the backbone mannopyranoside	500	Acid extraction	Immunomodulation	(18)
17	TP-2		Man, Xyl, GlcA in the ratio of 52.7:20.7:21.4	the backbone was composed of (1 → 3)-linked β-D-mannopyranoside, and the side chain composed of (1 → 6)-linked β-D-xylopyranoside was attached to the C2 of the backbone mannopyranoside	200	Acid extract	Immunomodulation	(18)
18	TP-3		Man, Xyl, GlcA in the ratio of 54.5:20.1:25.4	the backbone was composed of (1 → 3)-linked β-D-mannopyranoside, and the side chain composed of (1 → 6)-linked β-D-xylopyranoside was attached to the C2 of the backbone mannopyranoside	100	Acid extract	Immunomodulation	(18)
19	TP-4		Man, Xyl, GlcA in the ratio of 58.8:19.4:21.8	the backbone was composed of (1 → 3)-linked β-D-mannopyranoside, and the side chain composed of (1 → 6)-linked β-D-xylopyranoside was attached to the C2 of the backbone mannopyranoside	30	Acid extract	Immunomodulation	(18)
20	TP-5		Man, Xyl, GlcA in the ratio of 60.0:18.1:21.9	the backbone was composed of (1 → 3)-linked β-D-mannopyranoside, and the side chain composed of (1 → 6)-linked β-D-xylopyranoside was attached to the C2 of the backbone mannopyranoside	10	Acid extract	Immunomodulation	(18)
21	TP-6		Man, Xyl, GlcA in the ratio of 62.7:17.3:19.8	the backbone was composed of (1 → 3)-linked β-D-mannopyranoside, and the side chain composed of (1 → 6)-linked β-D-xylopyranoside was attached to the C2 of the backbone mannopyranoside	4	Acid extract	Immunomodulation	(18)
22	TPS			Not reported	582	Hot-water extraction	Antioxidant	(66)
23	TL04		Rha, Man, GlcA in the ratio of 1.0:5.0:1.9	The backbone is composed of (1 → 2)- and (1 → 4)-linked-Man and (1 → 3)-linked-glucans	2030	Hot-water extraction	Neuroprotective effect	(31)
24	TF-1			Not reported	3,430	Hot-water extraction		(59)
25	TF-2			Not reported	3,740	Hot-water extraction		(59)
26	TF-3			Not reported	3,740	Hot-water extraction		(59)
27	TFP	Acidic heteropolysaccharide	Man, GlcA, Xyl, Fuc = 6.8:1.0:1.5:0.6	a polysaccharide with Man as its main chain and GlcA, Fuc and Xyl as well as a small amount of Glc as the branch chain	1860	Hot-water extraction	Drug control, release	(58)
28	TP-1	Acidic heteropolysaccharide		Man as the main chain	8,160	Hot-water extraction		(57)
29	TP-2	Acidic heteropolysaccharide		Man as the main chain	11,200	High-pressure water extraction		(57)
30	TP-3	Acidic heteropolysaccharide		Man as the main chain	6,350	Alkali extraction		(57)
31	TP-4	Acidic heteropolysaccharide		Man as the main chain	3,550	Acid extraction		(57)
32	TP-5	Acidic heteropolysaccharide		Man as the main chain	11,600	Enzyme-assisted extraction		(57)
33	TPS20	Acidic heteropolysaccharide		Not reported	9,870	Hot-water extraction		(67)
34	TPS40	Acidic heteropolysaccharide		Not reported	8,640	Hot-water extraction		(67)
35	TPS60	Acidic heteropolysaccharide	Man, Fuc, Xyl, GlcA, Glc, Gal, Arab, Rha, Rib, GalA in the ratio of 871.7:324.0:251.0:218.0:15.5:4.0:2.0:1.7:1.0:0.7	Not reported	7,720	Hot-water extraction		(67)
36	TPS80	Acidic heteropolysaccharide		Not reported	6,010	Hot-water extraction		(67)
37	TFP-F1		Fuc, Xyl, Man, GlcA in the ratio of 0.9:1.0:3.2:1.2	{→3)-[β-D-GlcAp-(1 → 2)]-α- d - manp -(1 → 3)-*α*- d - manp -(1 → 3)-[α-L-Fucp-(1 → 2) β-D-Xylp-(1 → 2)]-α- d - manp -(1→} n	1870	Hot-water extraction	Immunomodulation	(16)
38	TFPS	Acidic heteropolysaccharide	Gal, Glc, Fru, Xyl, Fuc, Man in the ratio of 1.0:6.5:10.0:8.5:0.5:7.5	the backbone was Man to GlcA and Fuc side chains are attached	7	Hot-water extraction	Improve obesity	(41)

Glc, glucose; Fru, fructose; Gal, galactose; Rib, ribose; Xyl, xylose; Arab, arabinose; Man, mannose; GlcA, glucuronic acid; Rha, rhamnose; GalA, galacturonic acid; Fuc, fucose.

Kakuta et al. (64) isolated the acidic polysaccharides HWS-A and HAS-A through alkali extraction, followed by fractionation and purification, yielding approximately 70% of the total extractable material. The monosaccharide ratios of D-Xyl, D-Man, and D-GlcA were 1.0:2.9:1.0 for HWS-A and 1.0:4.9:1.3 for HAS-A. Ge et al. (65) isolated and purified the acidic heteropolysaccharide TFBP-A from *Tremella fuciformis* using DEAE-32 cellulose and Sephadex G-200 chromatography. The monosaccharide composition of TFBP-A was Man: Gal: Glc in a molar ratio of 90:5:5, with a molecular weight of 5.9 × 10^4^ Da. Gao et al. (21) isolated and purified three polysaccharides (T1a, T1b, and T1c) from TFPS using hot-water extraction, ethanol precipitation, and DEAE-Sephadex A-50 chromatography. The molecular weights of T1a, T1b, and T1c were 5.3 × 10^4^, 1.8 × 10^4^, and 1.8 × 10^4^ Da, respectively. Acidic hydrolysate fractions of T1a (T1a-1, 2, 3, 4, 5), with molecular weights ranging from 1.0 × 10^3^ to 5.3 × 10^4^ Da, were capable of inducing interleukin-6 (IL-6) secretion in monocytes, similar to the parent compound T1a. Additionally, Gao et al. (19) also isolated four polysaccharides (T2a, T2b, T2c, and T2d) from *Tremella fuciformis*, with molecular weights of 4.1 × 10^5^, 2.5 × 10^5^, 3.4 × 10^4^, and 2.0 × 10^4^ Da, respectively. Gao et al. (20) isolated four acidic polysaccharides from *Tremella fuciformis* by aqueous extraction (T3a, T3b, T3c, and T3d), with relative molecular masses of 5.5 × 10^5^, 4.2 × 10^5^, 5.5 × 10^4^, and 4.8 × 10^4^ Da, respectively. T3a-T3d were shown to induce the production of IL-1, IL-6, and tumor necrosis factor (TNF) in human monocytes *in vitro*. Acid hydrolysate fragments of T3a (T3a-1, T3a-2, T3a-3, T3a-4, and T3a-5A) also efficiently induced IL-6 secretion in monocytes. Further purification of *Tremella fuciformis* polysaccharide (WTF-B) using DEAE-Sephadex A-25 and Sephadex G-200 chromatography yielded a radioprotective, water-soluble homogeneous polysaccharide. WTF-B consisted of Glc and Man in a molar ratio of 8:2, with a molecular weight of 6.8 × 10^4^ Da (43). Jiang et al. (18) obtained six fractions of TFPs (TP-1 to TP-6) by hydrolyzing crude TFPs. The average molecular weights of TP-1 to TP-6 were 5.0 × 10^5^ Da, 2.0 × 10^5^ Da, 3.0 × 10^4^ Da, 1.0 × 10^5^ Da, 1.0 × 10^4^ Da, and 4.0 × 10^3^ Da, respectively. The physicochemical properties, monosaccharide composition, and structural analysis of TP-1 through TP-6 revealed similar repeating unit structures. Liu et al. (66) isolated the primary purified polysaccharide fraction (TPS) from *Tremella fuciformis* seeds using DEAE-52 and Sepharose CL-4B chromatography. The molecular weight of TPS was 5.8 × 10^5^ Da. Jin et al. (31) isolated the neuroprotective *Tremella fuciformis* polysaccharide TL04 (molecular weight: 2.0 × 10^6^ Da), a heteropolysaccharide composed of Rha, Man, and Glc in a molar ratio of 1.0:5.0:1.9, using hot-water extraction, freeze-drying, and DEAE-52 cellulose anion-exchange followed by Sepharose G-100 chromatography. Wu et al. (59) used asymmetric flow field-flow fractionation to analyze high-molecular-weight branched polysaccharides, isolating three polysaccharides (TF1, TF2, and TF3) from *Tremella fuciformis* via hot-water extraction, with molecular weights of 3.4 × 10^6^, 3.7 × 10^6^, and 3.7 × 10^6^ Da, respectively. Wang et al. (58) isolated and purified an acidic polysaccharide (TFP) from *Tremella fuciformis*, which was assembled into a nanostructure with chitosan for controlled release. TFP, with a molecular weight of 1.9 × 10^6^ Da, is composed of Man, GlcA, Xyl, and Fuc in a molar ratio of 6.8:1.0:1.5:0.6. Wang et al. (57) employed various methods to extract crude polysaccharides (TPs) from *Tremella fuciformis*, including hot-water extraction (TP1), high-pressure water extraction (TP2), alkali extraction (TP3), acid extraction (TP4), and enzyme-assisted extraction (TP5). The molecular weights of crude polysaccharides extracted from *Tremella fuciformis* were 8.2 × 10^6^, 1.1 × 10^7^, 6.4 × 10^6^, 3.6 × 10^6^, and 1.2 × 10^7^ Da, respectively. Lan et al. (67) fractionated *Tremella fuciformis* polysaccharides into TPS20, TPS40, TPS60, and TPS80 through stepwise ethanol precipitation. The viscosities of TPS20, TPS40, and TPS60 increased when the sucrose concentration was below 6%. Strongly acidic and strongly alkaline conditions reduced the viscosity of TPS solutions. Huang et al. (16) isolated a bioactive polysaccharide, TFP-F1, with a high molecular weight of 1.9 × 10^6^ Da from *Tremella fuciformis*. Monosaccharide composition and NMR analysis revealed that the polysaccharide and its derivatives contained Fucp, Xylp, Manp, and GlcAp in a molar ratio of 0.9:1.0:3.2:1.2. Chiu et al. (41) purified and fractionated an ameliorated-obesity polysaccharide from *Tremella fuciformis* using anion-exchange chromatography on a DEAE-650 M column. TFPS, with a molecular weight of 6.8 × 10^3^ Da, is a heteropolysaccharide composed of Gal, Glc, Fru, Xyl, Fuc, and Man in a molar ratio of 1.0:6.5:10.0:18.5:30.5:67.5, as determined by high-performance size-exclusion chromatography (HP-SEC).

The chemical structure of TFPS is primarily composed of a linear 1,3-linked *α*-D-Man backbone with highly branched *β*-D-Xyl, α-D-Man, and β-D-GlcA side chains. The common monosaccharides include Man, Xyl, Fuc, Glc, Gal, and GlcA (59, 68). However, Ma et al. (69) suggested that TFPs may not be restricted to the previously reported skeletal structure with Man as the backbone. Additionally, Jin et al. (31) purified the *Tremella fuciformis* polysaccharide TL04 from its aqueous extract. The backbone of TL04 consists of (1 → 2)- and (1 → 4)-linked Man, as well as (1 → 3)-linked glucans.

Although TFPs contain potential bioactive and functional components, their chain conformation and physicochemical properties remain poorly understood. Beyond molecular weight and monosaccharide composition, limited information is available regarding the structure and conformation of TFPs. Huang et al. (16) reported that the immunomodulatory polysaccharide TFP-F1, isolated from *Tremella fuciformis*, has a structure of [→3)-[*β*-D-GlcAp-(1 → 2)]-*α*-D-Manp-(1 → 3)-α-D-Manp-(1 → 3)-[α-L-Fucp-(1 → 2)-β-D-Xylp-(1 → 2)]-α-D-Manp-(1→]_n_, with partial C6-OH acetylation on Man residues. Additionally, TFP-F1 stimulated TNF-α and IL-4 secretion in J1A.4 macrophages *in vitro* by interacting with Toll-like receptor 6 (TLR6) at a concentration of 1 μg/mL. Removal of the O-acetyl group abolished immunomodulatory activity, suggesting its critical role in enhancing pro-inflammatory cytokine production.

Jiang et al. (18) isolated six immunomodulatory polysaccharides (TP-1 to TP-6) from *Tremella fuciformis* through water decoction, hydrolysis with 0.1 mol/L hydrochloric acid, and Sephadex G-150 column chromatography. TFPs increased peripheral blood leukocyte counts reduced by cyclophosphamide, with lower molecular weight fractions exhibiting superior protective effects. Additionally, TP-1 to TP-6 were homogeneous, with monosaccharide components including Man, Xyl, and GlcA, among which Man was the most abundant. Their structures featured nonreducing *β*-D-glucopyranuronic terminals, a backbone of (1 → 3)-linked β-D-mannopyranoside, and a side chain of (1 → 6)-linked β-D-xylopyranoside attached to the C2 position of the backbone mannopyranoside. The structures of TP-1 to TP-6 are consistent with those of *T. fuciformis* polysaccharides, which implies that TFP_S_ have repeating units can be prepared by acid hydrolysis.

The structural characteristics of polysaccharides isolated from *Tremella fuciformis* were investigated using ethanol precipitation and ion exchange chromatography, followed by HPGPC, HPLC, GC–MS, methylation (MT) analysis, infrared (IR), and nuclear magnetic resonance (NMR) spectroscopy (7). The NMR signals were predominantly observed between 20 and 180 ppm. The primary components included Man, GlcA, Glc, Gal, Xyl, and Rha. *δ* 80.6, 79.8, and 79.3 likely correspond to C4 of → 4)-Xylp-(1→, → 4)-Galp-(1→, and → 4)-Man-(1→, respectively, while δ 76.8 may correspond to → 4)-Glcp-(1→. Furthermore, δ 68.3 and 67.2 shifted to the low field, suggesting C6 substitution. This indicates the possible presence of C6-substituted glycosidic bonds in → 6)-Galp-(1→, → 4,6)-Manp-(1→, → 3,6)-Galp-(1→, and → 3,4,6)-Manp-(1→. The primary linkage types were identified as 1,4-Xylp, 1,4-Manp, 1-Xylp, 1-Manp, 1,4-Glcp, and 1,3,4-Galp.

## 4 Pharmacological action of TFPs

**Figure 2:**
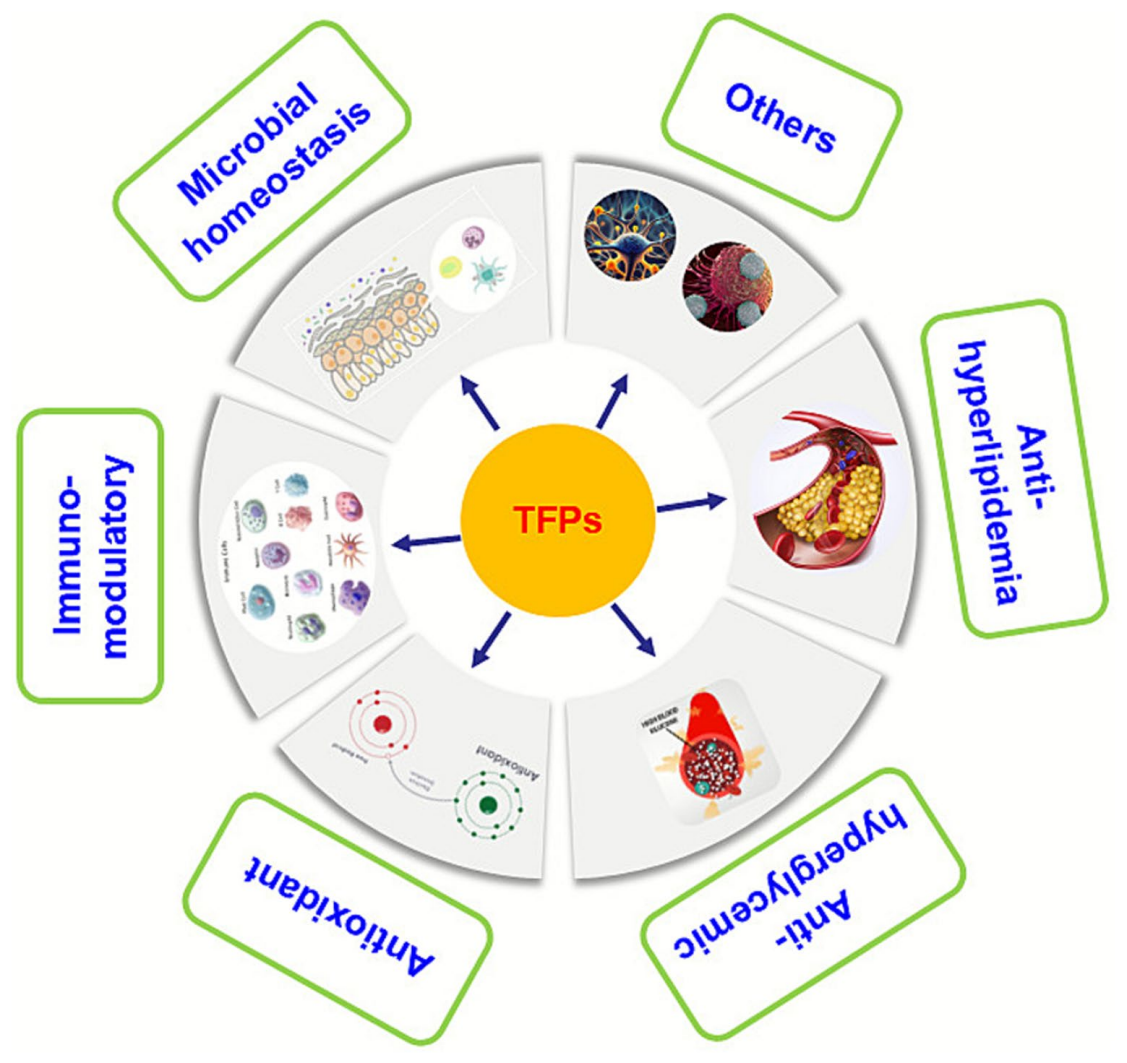
The pharmacological effects of the TFPs.

Recently, numerous *in vitro* and *in vivo* studies have been conducted to explore the various biological activity and mechanism of action of TFPs, including microbial homeostasis, immuno-modulatory, antioxidant, anti-hyperglycemic, anti-hyperlipidemia, and others (Figure 2). These biological activities and health benefits of TFPs will be discussed one by one in the following paragraphs.

### 4.1 Regulation effect of TFPs on microbial homeostasis

Due to the lack of carbohydrate-active enzymes (CAZymes) in the human body, most polysaccharides cannot be directly absorbed. The gastrointestinal tract serves as one of the primary sites for the functional expression of natural polysaccharides. Gut microbiota plays a crucial role by enhancing CAZyme activity, thereby degrading polysaccharides into monosaccharides or oligosaccharides and promoting the growth of probiotics.

**Figure 3:**
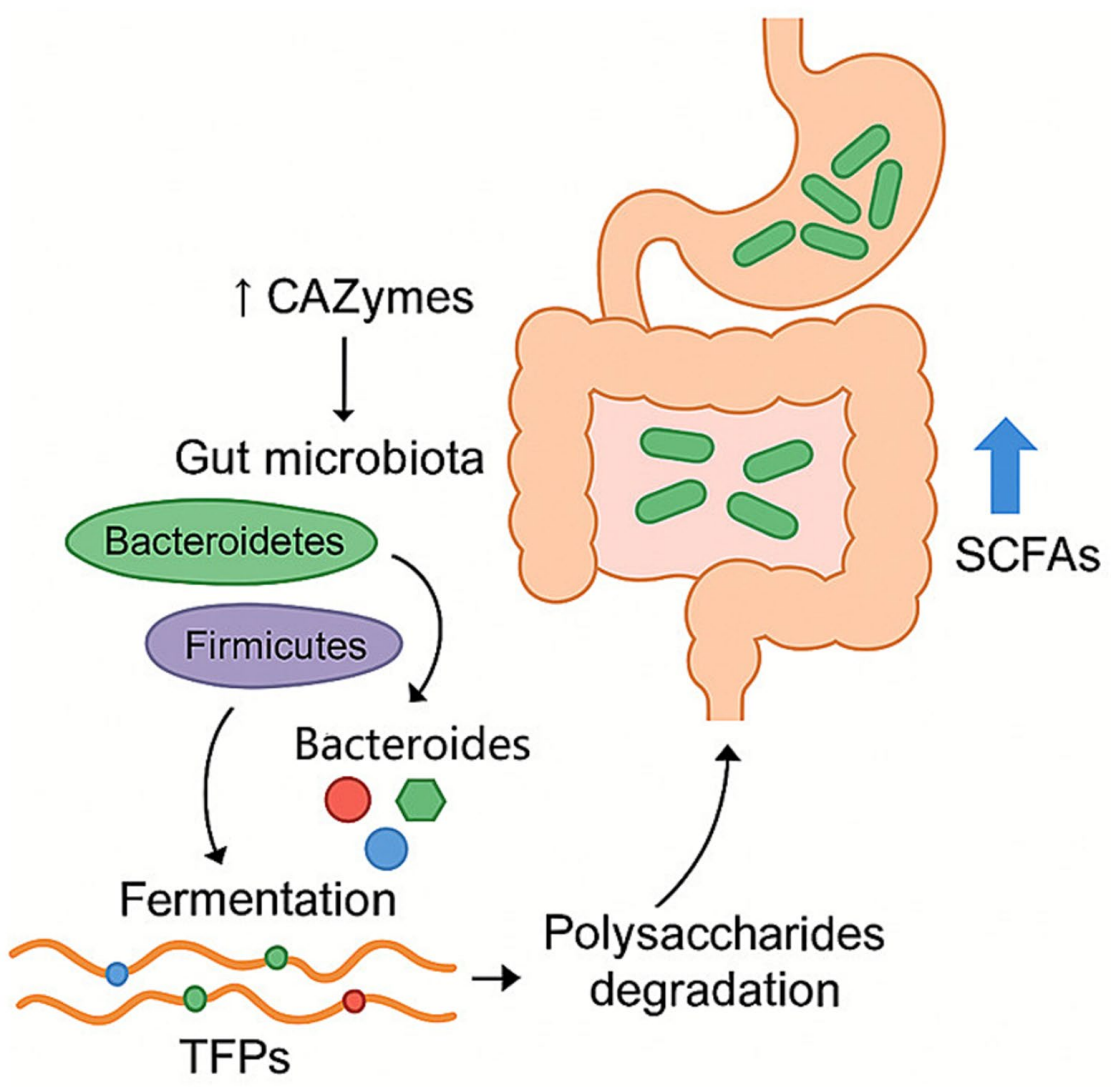
Regulation effect of TFPs on microbial homeostasis. TFPs are fermented by gut microbiota, particularly Bacteroidetes, which degrade them using CAZymes. This process promotes the proliferation of beneficial bacteria and leads to the production of SCFAs, thereby modulating the overall composition of the gut microbiome.

*Tremella fuciformis* polysaccharides can modulate the gut microbiota, where they are fermented by intestinal microbes, supporting microbial proliferation and increasing the production of short-chain fatty acids (Figure 3). Some reports show changing gut microbiota composition *in vitro* and *in vivo* of TFPs by fermentation. TFPs mainly affect the relative abundance of Firmicutes and Bacteroidetes (22, 26, 28, 40). In the *in vitro* fermentation environment of healthy volunteers, when the molecular weight of polysaccharide was less than 1800 kDa, the relative abundance of Bacteroides increased, and that of firmicutes decreased. When the molecular weight of polysaccharide was more than 1800 kDa, the relative abundance of Bacteroidetes and Firmicutes increased. Bacteroidetes was one of the main gut bacteria that were responsible for degrading polysaccharides. It released polysaccharide hydrolases and glycoside hydrolases for the degradation of the Gal side chain structures along with the backbone of TFPs. The pharmacological actions of TFPs on microbiome are shown in Table 2.

**Table 2:** Summarization of TFPs on gut microbiota.

Type	Molecular weight	Donor	Constituent monosaccharides and molar ratios	Changes in microbiota
TFPs (72)	16.62 ± 0.16 kDa	Healthy volunteers 18 to 25 years	Man: GlcA: Glc: Xyl: Fuc = 1.00:0.07:0.58:0.41:0.20	*Megasphaera*, *Phascolarctobacterium*, and *Bacteroides*↑ *Escherichia-Shigella* and *Fusobacterium*↓
TFPs (73)	~1184.50 kDa	Healthy specific pathogen free rat	Man: GlcA: Glc: Xyl: Fuc = 1.00:0.16:0.09:0.47:0.38	*Parabacteroides* and *Bacteroides*↑; SCFAs↑ *Eubacterium*↓
TFPs (26)	1855.60 ± 20.40 kDa	Healthy volunteers 18 to 26 years	Man: GlcA: Glc: Xyl: Fuc = 1.00:0.08:0.02:0.31:0.20	*Bacteroides*, *Phascolarctobacterium*, and *Lachnoclostridium*↑; SCFAs↑ *Fusobacterium*, *Klebsiella*, *Morganella*, *Bilophila*, *Escherichia-Shigella*↓
TFPs (40)	10.07 to 66.29 kDa	HFD-fed mice	Man: Rha: GlcA: Glc: Gal: Xyl = 1.00:0.02:0.15:0.08:0.20:0.11	*Desulfovibrio*, *Bilophila*, *Blautia*, and *Intestinimonas*↑; SCFAs↑ *Muribaculaceae*, *Lachnospiraceae_NK4A136_group*, *Alistipes*, *Colidextribacter*, *Lactobacillus*↓
TFPs (28)	246.00 to 305.00 kDa	DSS-treated mice	Man: GlcA: Glc: Xyl: Fuc = 1.00:0.30:0.02:0.02:0.33:0.26	*Lactobacillaceae* and *Lactobacillus*↑; SCFAs↑ *Odoribacter*, *Helicobacter*, *Ruminococcaceae*, and *Marinifilaceae*↓
TFPs (22)	30.00 to 1230.00 kDa	Dinitrofluorobenzene-induced allergic dermatitis in mice	Man: Glc: Arab: Xyl = 1.00:0.29:0.29:0.38	*Alloprevotella* and *Prevotellaceae-UCG-001*↑; SCFAs↑ *Acetitomaculum*, *Family XIII AD3011_group* and *Clostridia_UCG-014*↓

Glc, glucose; Fru, fructose; Gal, galactose; Xyl, xylose; Arab, arabinose; Man, mannose; GlcA, glucuronic acid; Rha, rhamnose; Fuc, fucose.

### 4.2 Regulation effect of TFPs on immunity

**Figure 4:**
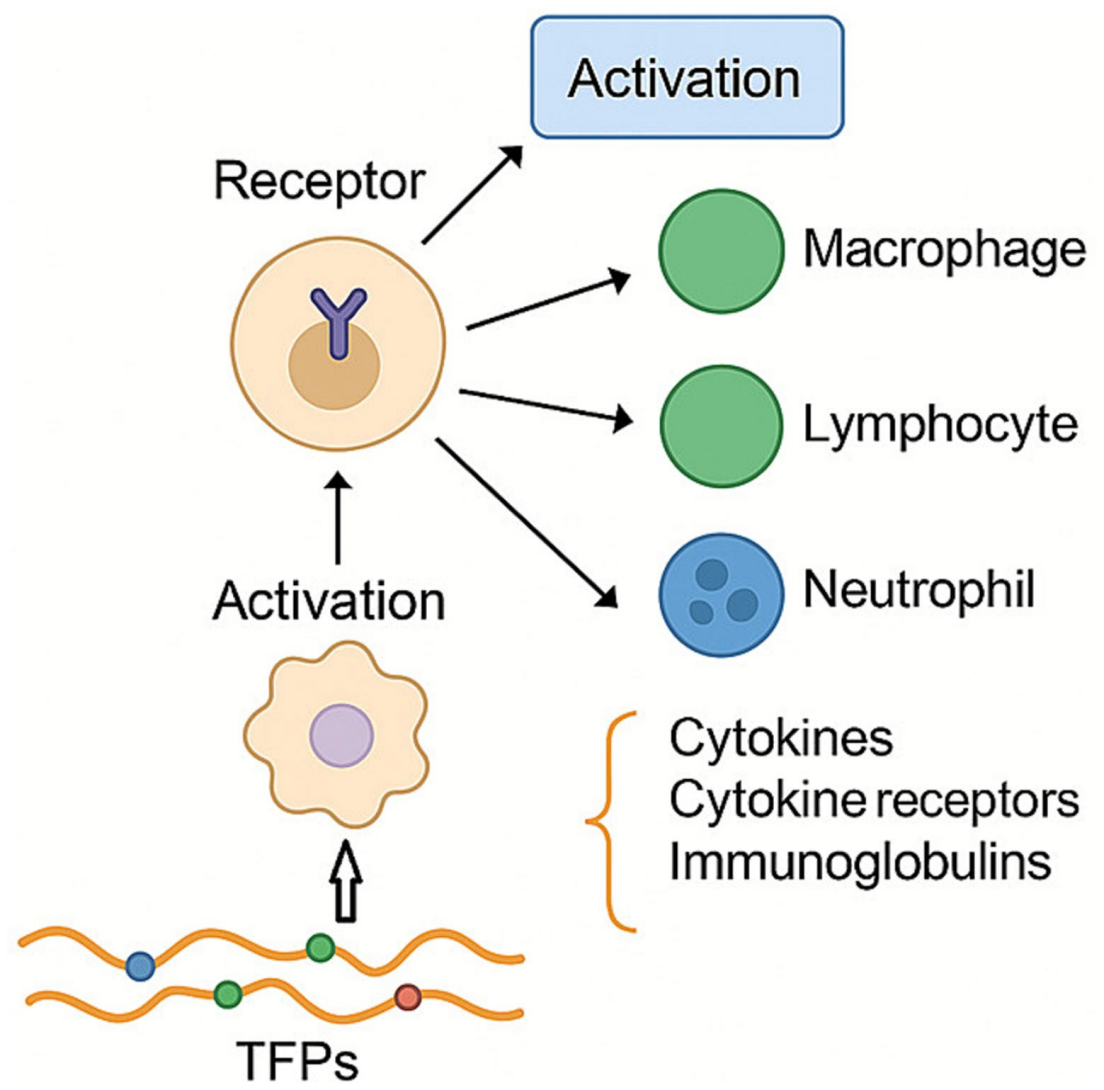
Regulation effect of TFPs on immunity. TFPs exert their effects by binding to immune cell receptors, leading to cell activation and the balanced secretion of cytokines.

Studies have shown that plant polysaccharides exert their effects by binding to specific receptors on the surface of macrophages, thereby triggering their activation (Figure 4). TFPs not only activate various immune cells-such as macrophages, lymphocytes, neutrophils, and natural killer cells-but also stimulate the production of immune mediators, including cytokines, cytokine receptors, and immunoglobulins.

*Tremella fuciformis* polysaccharides enhance cyclophosphamide-induced immunosuppression by reducing pro-inflammatory cytokines and increasing anti-inflammatory cytokines (17). TFPs can reverse cyclophosphamide-induced reductions in peripheral blood leukocyte counts in rats. Notably, TFPs with lower molecular weight exhibits higher immune response (18). Xie et al. showed that both oral and topical TFPs alleviated symptoms of dinitrofluorobenzene-induced atopic dermatitis in mice by reducing skin barrier dysfunction, ear edema, and epidermal thickening, with oral administration proving more effective (22). TFPs treatment suppressed the activation of p38MAPK pathway, alleviated oxidative stress, reduced inflammatory cytokines, and scavenged reactive oxygen species (ROS) in LPS-stimulated RAW264.7 macrophages (23). Additionally, TFPs reversed Treg-mediated immune suppression and reduced mortality in burn-induced septic mice by downregulating IL-10 and promoting Th1-to-Th4 transition (25).

Gao et al. (19–21) isolated 11 heteropolysaccharides (T1a-c, T2a-d, and T3a-d) from *Tremella fuciformis*, which induced IL-1, IL-6, and TNF production in human monocytes *in vitro*. Acidic hydrolysis products of T1a (T1a-1, T1a-2, T1a-3, T1a-4, and T1a-5) induced IL-6 production in human monocytes. Degradation products of T2a and T2b (T2a-S, T2a-L, and T2b-D) efficiently induced IL-1 secretion in monocytes. Various fragments of T3a acidic hydrolysate (T1a-2, T1a-3, T1a-4, T1a-5, and T1a-6a) induced IL-6 secretion in monocytes. The common (1 → 3)-mannan structure of the three heteropolysaccharides and their fragments likely underpins the immunomodulatory activity of TFPs, with molecular weight having no significant effect.

### 4.3 Regulation effect of TFPs on antioxidant system

**Figure 5:**
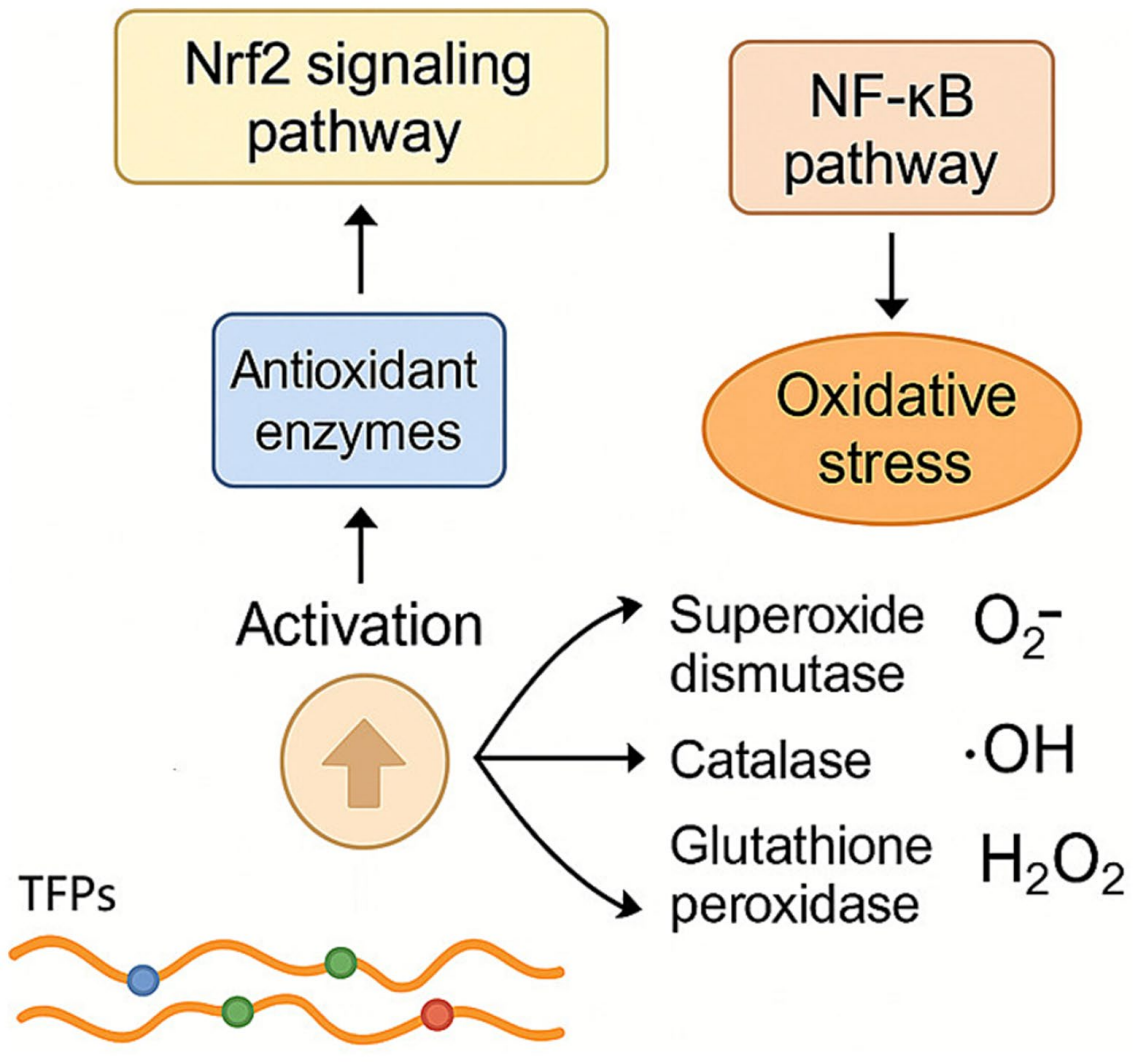
Regulation effect of TFPs on antioxidant system. TFPs bolster cellular antioxidant defense by upregulating key enzymes (e.g., SOD, CAT) through the Nrf2 pathway, while simultaneously directly neutralizing various ROS. They also inhibit the NF-κB pathway to reduce subsequent inflammation.

TFPs have demonstrated significant antioxidant regulatory functions through multiple mechanisms (Figure 5). TFPs enhance the activity of key antioxidant enzymes, including superoxide dismutase, catalase, and glutathione peroxidase, boosting cellular resistance to oxidative stress (6). They also activate the nuclear factor erythroid 2-related factor 2 signaling pathway, promoting the expression of downstream antioxidant enzymes, while suppressing oxidative stress-induced inflammation via inhibition of the nuclear factor kappa B pathway. TFPs also effectively scavenge ROS, such as superoxide anions (O₂^−^·), hydroxyl radicals (·OH), and hydrogen peroxide (H₂O₂), reducing oxidative damage (7, 8). Low molecular weight TFPs demonstrated strong hydroxyl radical scavenging activity, indicating that reducing molecular weight may enhance their overall antioxidant potential. These multifaceted mechanisms suggest that TFPs hold potential for preventing and treating oxidative stress-related diseases, such as neurodegenerative disorders, cardiovascular diseases, and diabetes. However, further research is needed to elucidate their precise molecular targets and dose–response relationships.

### 4.4 Regulation effect of TFPs on lipid metabolism, blood glucose, and insulin resistance

**Figure 6:**
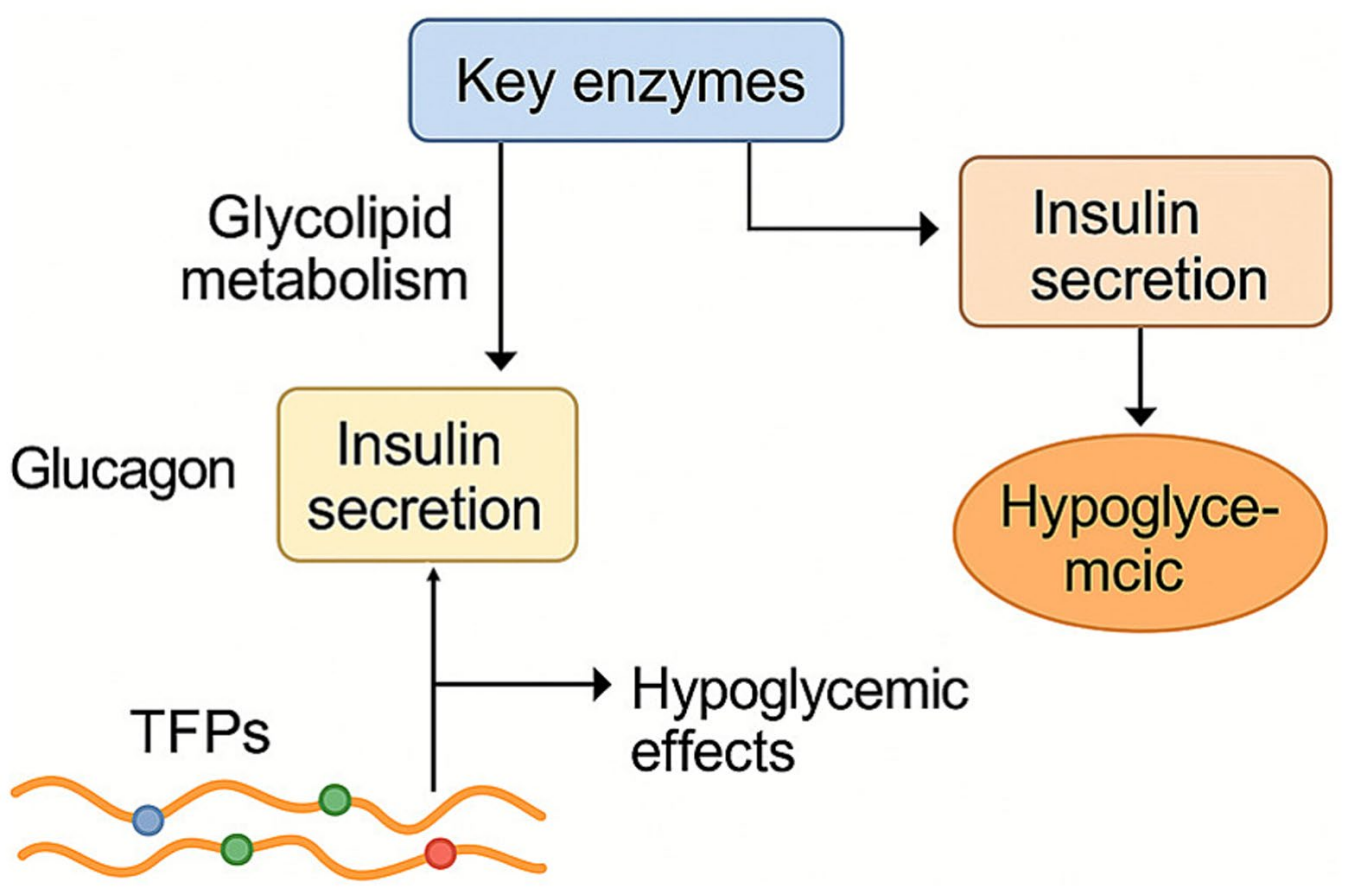
Regulation effect of TFPs on lipid metabolism, blood glucose, and insulin resistance. TFPs alleviate hyperglycemia by stimulating insulin secretion and enhancing hepatic glucose metabolism. Concurrently, they reduce lipid accumulation by suppressing the expression and activity of key lipogenic enzymes.

*Tremella fuciformis* polysaccharides can regulate key enzymes related to glycolipid metabolism and antagonize the elevation of glucagon (Figure 6). Additionally, TFPs promote insulin secretion, modulate the rates of glycogen synthesis and degradation, and contribute to the repair of damaged pancreatic islet cells, thereby exerting hypoglycemic effects. TFPs supplementation significantly alleviated weight gain, fat accumulation, hyperglycemia, and hyperlipidemia induced by high-fat diet (40, 41). Khan et al. (29) evaluated the effects of *Tremella fuciformis* crude polysaccharide (TFCP) on lipid profiles. TFCP significantly inhibited hepatic lipid accumulation, and enzymes responsible for fat acid synthesis (including HMG-CoA reductase, acetyl-CoA carboxylase, and fatty acid synthase). TFPs treatment significantly also activated peroxisome proliferator-activated receptor *γ*, a key regulator of insulin action (38). Additionally, glucuronide mannans from *T. fuciformis* seeds exhibited strong hypoglycemic effects, enhancing insulin secretion and hepatic glucose metabolism (39). The structure of glucuronide mannans is closely associated with its biological function, as glucuronide mannans with removed side chains was less active than natural glucuronide mannans.

### 4.5 Other pharmacological action of TFPs

A growing body of research highlights the neuroprotective and cognitive-enhancing effects of TFPs. In a randomized, double-blind, placebo-controlled trial, Ban et al. demonstrated that TFPs supplementation significantly improved subjective memory complaints, short-term memory, and executive function in individuals with cognitive impairment, accompanied by increased gray matter volume (30). TFPs also improves cognitive function by enhancing hippocampal CREB-positive neurons, glucose uptake, and cholinergic neurons (33, 35). Similarly, TFPs reversed scopolamine-induced memory deficits and promoted axonal growth and cholinergic activity (34).

The anti-tumor activity of TFPs is primarily manifested through the promotion of tumor cell apoptosis and immunostimulatory effects. Li and Xie et al. (13, 14) found that TFPs promoted apoptosis of B16 melanoma cells by inducing G2/M cell cycle arrest, and activating lipid transport and metabolism. TFPs can induces melanoma cell apoptosis by promoting M1 macrophage polarization (14). Han et al. explored the immunomodulatory and antitumor properties of polysaccharides derived from various *Tremella fuciformis* strains (12). Hot-water-extracted TFPs significantly increased mRNA levels of inducible nitric oxide synthase, IL-6, and tumor necrosis factor-*α* (TNF-α). Compared to hot water extracts, cold water-extracted TFPs exhibit stronger inhibitory effects on cancer cell viability and more effectively induce apoptosis.

## 5 Structure–activity relationship of TFPs

Polysaccharides with diverse biological activities exhibit substantial variation in their chemical composition, conformation, and physicochemical properties. However, elucidating the structure–activity relationships of TFPs remains challenging due to limited research. Nonetheless, several correlations have been reported.

Huang et al. characterized TFP-F1 with the structure [→3)-[*β*-D-GlcAp-(1 → 2)]-*α*-D-Manp-(1 → 3)-α-D-Manp-(1 → 3)-[α-L-Fucp- (1 →2)-β-D-Xylp-(1 → 2)]-α-D-Manp-(1→]_n_, partially acetylated at C6-OH of Man (16). At 1 μg/mL, TFP-F1 induced TNF-α and IL-6 production in J774A cells and promoted macrophage apoptosis via TLR4 signaling. Deacetylation abolished its immunomodulatory activity, highlighting the critical role of O-acetyl groups in cytokine induction.

Uronic acids are essential for TFPS bioactivity. Wu et al. evaluated five TFPs (TFP-I, TFPI-6, TFPI-12, TFPI-24, and TFPI-48), reporting that during human fecal fermentation, total polysaccharides declined from 91.19 to 65.95%, while uronic acid content decreased from 6.79 to 5.66% (26). TFP-I degradation accelerated between 12 and 48 h, with utilization reaching 53.76%, suggesting effective microbial fermentation and preferential use of uronic acids by colonic microbiota. Chiu et al. found that TFPs significantly mitigated HFD-induced obesity in mice (41). This effect was attributed to TFPs’ high viscosity and uronic acid content (26.7%), as uronic acids like galacturonic and glucuronic acids can bind cholesterol, potentially reducing lipid accumulation (70).

Chemical modification further enhances TFPS bioactivity. Sulfated TFPs show improved immunostimulatory, antiviral, and antioxidant properties, underscoring the role of structural modifications in optimizing biological function. Wang et al. synthesized carboxymethylated polysaccharides (CATP) from insoluble crude TFPS (71). Increased degrees of substitution (DS) improved water solubility, antioxidant, and moisturizing activities. Similarly, Liu et al. (68) reported that catechin-grafted TFPs exhibited enhanced thermal stability, crystallinity, and radical-scavenging capacity (66). These findings indicate that functional group grafting can substantially improve the antioxidant properties of TFPs, supporting their potential in food and pharmaceutical applications.

## 6 Summary

*Tremella fuciformis* has been traditionally used for thousands of years as a tonic, body builder, and anti-inflammatory agent. Polysaccharides, the primary bioactive components of *Tremella fuciformis*, have gained global attention for their molecular weight, monosaccharide composition, TPFs side chain positions, and correlations with physiological functions. Numerous studies have demonstrated that *T. fuciformis* polysaccharides possess diverse biological activities, including anti-tumor effects, antioxidant properties, immune regulation, anti-inflammatory actions, gastric protection, hepatoprotection, neuroprotection, hypoglycemic effects, radiation shielding, and drug delivery capabilities. However, most research on *Tremella fuciformis* polysaccharides is limited to preclinical studies using *in vivo* and *in vitro* animal models. The higher-order structure of TPF active components and their relationships with biological activities remain unclear. Future research should focus on clinical validation, structure–function relationships, gut microbiota interactions, and standardized production processes to support the development of TPF-based functional foods and therapeutics.
